# Sensory Feedback in Hand Prostheses: A Prospective Study of Everyday Use

**DOI:** 10.3389/fnins.2020.00663

**Published:** 2020-07-07

**Authors:** Ulrika Wijk, Ingela K. Carlsson, Christian Antfolk, Anders Björkman, Birgitta Rosén

**Affiliations:** ^1^Department of Translational Medicine, Faculty of Medicine, Lund University, Malmö, Sweden; ^2^Skåne University Hospital, Lund, Sweden; ^3^Department of Biomedical Engineering, Faculty of Engineering, Lund University, Lund, Sweden

**Keywords:** artificial arm, amputation, amputation stumps, sensory feedback, upper limb, traumatic amputation

## Abstract

**Introduction:**

Sensory feedback in hand prostheses is lacking but wished for. Many amputees experience a phantom hand map on their residual forearm. When the phantom hand map is touched, it is experienced as touch on the amputated hand. A non-invasive sensory feedback system, applicable to existing hand prostheses, can transfer somatotopical sensory information via phantom hand map. The aim was to evaluate how forearm amputees experienced a non-invasive sensory feedback system used in daily life over a 4-week period.

**Methods:**

This longitudinal cohort study included seven forearm amputees. A non-invasive sensory feedback system was used over 4 weeks. For analysis, a mixed method was used, including quantitative tests (ACMC, proprioceptive pointing task, questionnaire) and interviews. A directed content analysis with predefined categories *sensory feedback from the prosthesis, agency, body ownership, performance in activity*, and *suggestions for improvements* was applied.

**Results:**

The results from interviews showed that sensory feedback was experienced as a feeling of touch which contributed to an experience of completeness. However, the results from the questionnaire showed that the sense of agency and performance remained unchanged or deteriorated. The ability to feel and manipulate small objects was difficult and a stronger feedback was wished for. Phantom pain was alleviated in four out of five patients.

**Conclusion:**

This is the first time a non-invasive sensory feedback system for hand prostheses was implemented in the home environment. The qualitative and quantitative results diverged. The sensory feedback was experienced as a feeling of touch which contributed to a feeling of completeness, linked to body ownership. The qualitative result was not verified in the quantitative measurements.

**Clinical Trial Registration:**

Name: Evaluation of a Non-invasive Sensory Feedback System in Hand Prostheses. Date of registration: March 15, 2019. Date the first participant was enrolled: April 1, 2015. ClinicalTrials.gov Identifier: NCT03876405 ORCID ID: https://orcid.org/0000-0002-4140-7478.

## Introduction

Amputation of a hand results in the loss of motor and sensory functions, but also changed body balance and self-esteem as well as a feeling of being mutilated (Murray and Forshaw, 2013). Impairments, activity limitations, and participation restrictions (World Health Organization, 2001) can all be consequences of the amputation. In the human hand there is a delicate interaction between motor and sensory functions which is important for good hand function and also for incorporating the hand in the body representation (Gardner and Johnson, 2013). Hand sensibility is crucial for motor performance and motor learning (Kandel et al., 2013). However, to execute a voluntary movement and to learn how to improve performance, several senses can be used. For example, amputees with myoelectric prostheses often use audio information from the motor of the prosthesis to help adjust the grip (Markovic et al., 2018b). Amputees also get some useful sensory information through vibrations in the socket when using the grip (Childress, 1980). An expected advantage of sensory feedback is to make the prosthesis easier to use and improve the body image and thus make social interaction easier (Ackerley and Kavounoudias, 2015). A concept of importance in prosthesis use is the sense of agency, which is the experience of causing a movement generated by motor commands. One way of documenting a sense of agency is by asking if the person had control over the movement (Kalckert and Ehrsson, 2012; Haggard, 2017). Today’s prostheses allow the user to feel agency concerning the prosthesis, but the lack of sensory feedback seems to be an important factor limiting the experience of body ownership of the prosthesis (Wijk and Carlsson, 2015).

The need of sensory feedback in prostheses is debated, but several recent studies have found that it is something that prosthesis users desire in their hand prostheses (Pylatiuk et al., 2007; Wijk and Carlsson, 2015; Benz et al., 2016; Farina and Amsuss, 2016), in addition to comfort, function, appearance, and durability (Biddiss et al., 2007; Cordella et al., 2016). Even if the performance in grasping tasks already is good, feedback could be beneficial for complex tasks and for situations when visual feedback is constrained. Regardless of the possible improvement in performance, the subjective experience of embodiment tends to increase when feedback is added (Markovic et al., 2018a). In addition, some studies have reported reduced PLP when sensory feedback is added to a prosthetic hand (Dietrich et al., 2012; Page et al., 2018; Petrini et al., 2018). In recent years researchers have tried to provide sensory feedback in hand prostheses in different ways (Schofield et al., 2014; Svensson et al., 2017; Pasluosta et al., 2018; Stephens-Fripp et al., 2018), using invasive methods, using implanted neural interfaces (Ortiz-Catalan et al., 2014; Oddo et al., 2016; Schiefer et al., 2016; Graczyk et al., 2018; Petrini et al., 2019), and using non-invasive methods through vibrotactile or mechanotactile feedback methods (Hebert et al., 2014; Clemente et al., 2016; Raveh et al., 2018; Schoepp et al., 2018). Studies with sensory prosthetic hands in home use are infrequently presented, but a few case reports are published (Ortiz-Catalan et al., 2014; Clemente et al., 2016; Graczyk et al., 2018; Cuberovic et al., 2019).

Schofield et al. (2014) illustrate three aspects of sensory feedback in hand prostheses. Feedback can be *somatopically matched* (the feedback is perceived as originating from the “correct” body part), *modality matched* (the sub-modality is matched, e.g., pressure is pressure) and *sensory substitution* by input from another sense (e.g., vision, hearing, vibrotactile or electrotactile feedback).

The non-invasive method used here provides somatotopically matched sensory feedback by use of the areas of referred sensation on the residual arm, that is, the PHM. This map of the lost hand and fingers is evoked when touching specific areas of the skin of the residual arm (Ramachandran et al., 1992; Ramachandran and Hirstein, 1998) and in one study was found in a majority of participants with acquired hand amputation at the transradial level (Ehrsson et al., 2008). The PHM is highly individual; some have a very detailed map with several specific areas with referred sensation, while others have a smudged map or only experience few areas of the phantom hand (Bjorkman et al., 2016). When the PHM is stimulated with relevant feedback from the prosthesis, somatotopically matched information can be sent to the brain. Results from a fMRI study showed that stimulation of the finger areas in the PHM on the residual arm activated the same areas in the primary somatosensory cortex as stimulation of the fingers in an able-bodied control group (Bjorkman et al., 2012). Not all amputees experience a PHM (Ehrsson et al., 2008), but touch on predefined areas on the forearm can be learned to be associated to specific fingers (Wijk et al., 2019).

Antfolk et al. (2012, 2013a) have earlier presented a non-invasive sensory feedback concept utilizing the PHM that is also somatotopically matched as well as modality matched, regarding pressure (Antfolk et al., 2012, 2013a). Often prosthetic solutions are tested in a laboratory environment, but the need to evaluate sensory feedback in prostheses in real-life activities has been highlighted (Schofield et al., 2014). The aim of this study was to evaluate how forearm amputees experienced a non-invasive sensory feedback system used in daily life over a 4-week period.

## Materials and Methods

### Sample

Inclusion criteria: acquired unilateral transradial amputation, experiencing a PHM (minimum three finger areas), experience using a myoelectric prosthesis and ability to understand and speak Swedish. Exclusion criteria: psychiatric or cognitive disorders. Seven regional prosthetic centers in Scandinavia were asked to be involved in the recruitment of subjects during 2014–2018, and three centers participated. Nine individuals that met the inclusion criteria were contacted and asked for participation, and of these seven agreed to participate.

Median age was 49 years (range 42–72 years, 3 women and 4 men). Five had an amputation of the dominant side. Time since amputation was at median 13 years (range 1–36 years). Five reported PLP. Five presently used a myoelectric prosthesis (4 myoelectric VariPlus Speed hand; OttoBock and 1 I-limb; Össur). One participant used an AxonHook, OttoBock but had used a myoelectric prosthesis earlier, and 1 had a passive aesthetic prosthesis but had also used a myoelectric prosthesis earlier. The normal prosthesis use was 6–16 h/day, with a median of 11 h/day.

### Design

To achieve a broad understanding of the outcome of the intervention, several evaluation and analysis methods were used: qualitative deductive analyses of interviews as well as quantitative measures by use of a questionnaire and objective measurements (method triangulation) (Carter et al., 2014). A longitudinal design was used in a series of cases to illuminate subjective experience and illustrate objective changes over time from use of sensory feedback from a hand prosthesis in daily life.

### Experimental Setup

**FIGURE 1:**
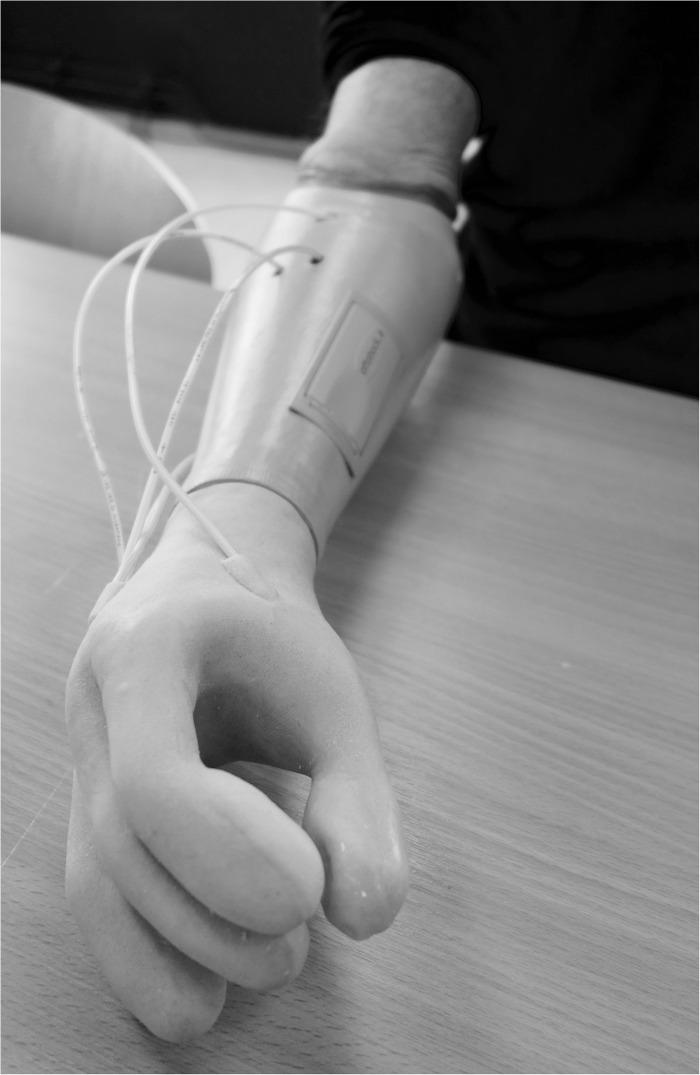
When the silicon bulbs in the fingertips were pressed, the air was transferred via plastic tubes that reached actuators inside the prosthetic socket and gave pressure (mechanotactile feedback) on the skin corresponding with the PHM zones.

A non-invasive air-mediated sensory feedback system (Antfolk et al., 2012, 2013b) was integrated in a prototype prosthesis glove. The sensory feedback system used was a simple non-invasive, non-electronic system based on air-mediated pressure, described by Antfolk et al. (2012). A silicone glove with bulbs (35 mm in length) volar in every fingertip was made and applied on a single degree-of-freedom prosthetic hand (VariPlus Speed hand, OttoBock), size 73/4. It had no wrist flexibility but manually adjustable wrist rotation. In Figure 1, the system is shown as integrated into a silicone glove. The sensing bulbs on the fingers were manually manufactured so there was some variation in their sizes due to manufacturing but also depending on in which finger they were positioned.

The stimulation given was mechanotactile and the pressure was transferred from the silicon bulbs in the fingertips of the prosthesis via plastic tubes that reached actuators (silicon bulbs 13 mm in diameter) inside the prosthetic socket. The pressure applied to the skin from the silicon bulbs depends on the force and speed at the fingertip level (Antfolk et al., 2012). The sensing bulb was roughly a half-cylinder with diameter 20 mm and length 35 mm. This gives a volume of roughly 5500 mm^3^ or 5.5 ml. The tubes were pneumatic tubes from FESTO (PUN-3 × 0.5 SI, FESTO, Esslingen am Neckar, Germany) with an inner diameter of 2.1 mm. In our previous paper (Antfolk et al., 2012), we measured the pressure generated by the sensing bulb using a pressure sensor when a monofilament was pressed against the sensing bulb. For a 60 g monofilament a pressure of 1.2 kPa was recorded, for a 100 g monofilament 2.3 kPa, 180 g monofilament 4.3 kPa, 300 g monofilament 6.5 kPa. More details on the sensing bulbs can be found in Svensson et al. (2020).

Other factors of importance for receiving the pressure are the quality of the skin (e.g., scarring) and damaged skin was avoided. The actuators were applied to the skin corresponding with the PHM areas (Figure 1).

Thus, it was possible to transfer both a modality matched and somatotopically matched feedback.

At the first meeting a “mapping” of the areas of the referred sensations in the residual forearm was done. The participants were asked to touch the skin on the residual forearm and define the zones with referred sensation of the PHM (digit I–V). The PHM was then marked with a pen, and the participant confirmed the mapping by blindly responding to stimulation of the different areas by the experimenter.

A casting for the prosthetic socket was made and the marks of the PHM were transferred to the inside of the socket. A socket with the sensory feedback system embedded was then constructed. When the participants were equipped with the prosthesis they were asked to orally confirm that the pressure was perceived, and somatotopically matched the PHM.

This was made through pressing the fingertips of the prosthetic hand. No structured training of the prosthetic hand or the sensory feedback was given.

The participants were asked to use the prosthesis at home for at least 2 h/day, 5 days/week over 4 weeks.

### Subjective Experiences From Questionnaire and Interviews

A questionnaire consisting of 21 statements concerning sensory feedback from the prosthesis, agency, body ownership, performance in activity, and PLP was developed by the first and last author. The questionnaire was based on the ones used in experiments of rubber hand illusion (Botvinick and Cohen, 1998; Ehrsson et al., 2008). In the questionnaire, the participants were asked to match each statement on a 7-level Likert scale from “Strongly disagree” (—) to “Strongly agree” (+++) (Botvinick and Cohen, 1998). Six control statements were included in the questionnaire, not related to the construct, aiming to capture suggestibility and task compliance.

After the test period, the first author carried out semi-structured interviews with open-ended questions. All participants were asked to describe the activities in which they used the prosthesis and how they experienced it, if and why they chose not to wear the prosthesis during some activities, the experiences of the sensory feedback from the prosthesis, agency and body ownership, and their suggestions for improving the feedback or the design of the prosthesis.

During the test period the participants were asked to keep a diary where they documented the time, activity, and place of wearing the prosthesis.

### Objective Outcome

Function/capacity was assessed with The Assessment of Capacity for Myoelectric Control (ACMC). This is a standardized observational assessment which rates the subjects’ capacity of myoelectric control in a bimanual activity, either standardized (such as “packing a suitcase for overnight stay,” “repotting a plant,” or “setting a table for four persons”) or self-chosen. The ACMC consists of 22 items and a 4-grade rating scale (0 = Not capable, 1 = Somewhat capable, 2 = Generally capable, 3 = Extremely capable). The ACMC units are calculated on the ACMC website, converted to interval-level linear measures by using the Rasch measurement analysis, and reported in a range of 0–100. The higher the score, the better the task performance^1^ (Lindner et al., 2009, 2014). In this study, the activity “to make a sandwich with toppings” was chosen by the authors. Actions included in the task are to take a bun from a bag, split it in two halves, spread butter on both halves, put cheese and ham, from a closed package, on the bread. Thereafter slice a tomato and cucumber and put on top. Wrap the sandwich in paper and clean up the table. The scoring was made by an experienced external rater (occupational therapist, certified ACMC-rater). The ACMC was performed without sensory feedback in the pre-tests and with sensory feedback at the follow up.

**FIGURE 2:**
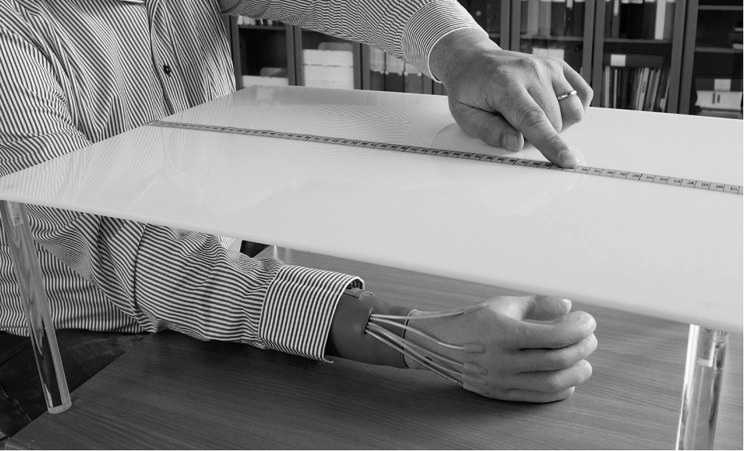
In the Proprioceptive pointing task the participants were asked, with their eyes closed, to mark on a ruler (proximally to distally from their own body) with their index finger on the sound hand, where they estimated the location of: (1) the prosthetic index finger and (2) where they experienced their phantom index finger.

Body ownership was assessed with the proprioceptive pointing task (Botvinick and Cohen, 1998; Rohde et al., 2011). The participants were asked to mark on a ruler (proximally to distally from their own body) with their index finger on the other hand and their eyes closed, where they estimated the location of the prosthetic index finger and where they experienced their phantom index finger (Figure 2).

The order in which the data collection was performed: Questionnaire, ACMC without feedback, proprioceptive pointing task, using the prosthesis with sensory feedback at home (4 weeks), questionnaire, ACMC with sensory feedback, proprioceptive pointing task, and interview. The tests were applied in one session before the test period and in one session for follow up, except for the interview that was only done at the follow up.

### Analysis

The interviews were transcribed by the first author and analyzed independently by the first and second authors (investigator triangulation) in a directed qualitative content analysis (Patton, 1999; Hsieh and Shannon, 2005) and according to the procedure described by Graneheim and Lundman (2004). The text was read and reread in order to obtain a sense of the entire data. Meaning units, that is, words or sentences related to the aim of the study, were then identified and thereafter coded while still preserving the core meaning (Table 1). Codes were then grouped into categories according to predefined concepts. The categories were then discussed with the other authors and adjustments were made to reach consensus.

**TABLE 1:** Example from the condensation in the directed content analysis.

Meaning unit	Code	Category
“When you grasp with it and feel that you grasp. It’s a fantastic feeling!”	Feel the grasp	Sensory feedback from the prosthesis

Regarding the authors’ preunderstanding, the first and second authors are occupational therapists with previous experience in qualitative research (Carlsson et al., 2010; Wijk and Carlsson, 2015). The third author is a researcher in biomedical engineering at the Department of Biomedical Engineering and has a long experience of research in the field of prosthetic hands; the fourth author is a hand surgeon; and the last author an occupational therapist. All authors except the third work at the Hand Surgery Department in Malmö, Sweden, and have long clinical experience (Graneheim and Lundman, 2004).

The quantitative results were analyzed and presented descriptively, added with Wilcoxon signed rank test to compare differences in pretests and follow-up regarding Questionnaire, ACMC, and Proprioceptive Pointing task. Results from the questionnaire were analyzed in the categories to predefined concepts (Table 2: sensory feedback, agency, body ownership, performance in activity, phantom limb pain). Graphpad Prism version 8.2.1 was used for calculation.

**TABLE 2:** Individual questions included in questionnaire and grouped according to concept.

Sensory feedback (SF)	SF1: “When I grip something it feels like I grasp it with my real fingers.” SF2: When I grip objects, I can feel the touch in the fingers of the prosthesis.”
Agency (AG)	AG1: “It feels like I control the movement of the prosthesis.” AG2: “The prosthesis moves like I want it to, like I am controlling it with my will.”
Body ownership (BO)	BO1: “It feels like the prosthesis is my hand.” BO2: “It feels like the prosthesis is a part of my body.” BO3: “It feels like the phantom hand is inside the prosthetic hand.”
Performance in activity (PIA)	PIA1: “I can use the prosthesis without looking.” PIA2: “I can put away a plastic cup without looking.” PIA3: “I can control the grip of the prosthesis.” PIA4: “I even feel that I can hold a small child with the prosthesis.” PIA5: “I feel that I can control how hard I hold something.” PIA6: “The prosthesis feels like a tool.”
Phantom limb pain (PLP)	PLP1: “I have phantom limb pain when wearing the prosthesis.” PLP2: “I have phantom limb pain when not wearing the prosthesis.”
Control questions	“It feels like the prosthesis controls my movements.” “My (real) arm feels rubbery when I wear the prosthesis.” “It feels like the prosthesis has its own will.” “My (real) arm feels like a robot when I use the prosthesis.” “Sometimes I perceive a feeling of touch somewhere outside the prosthesis.” “When I grip objects with the prosthesis, it feels like the feeling of touch is projected toward my upper arm and/or chest.”

## Results

The reported time the prosthesis with the sensory feedback system was used was 2–15 h/day. The wearing time for individual 1 to 7 was: 2–15 h, 2–3 h, 2 h, 2–5 h, 2 h, 2–4 h, 2–3 h, respectively. Since the wearing time were around 2–3 h for the majority of participants, no conclusions could be drawn regarding prosthesis use in relation to user experience or performance.

### Subjective Experiences

#### Questionnaire

**FIGURE 3:**
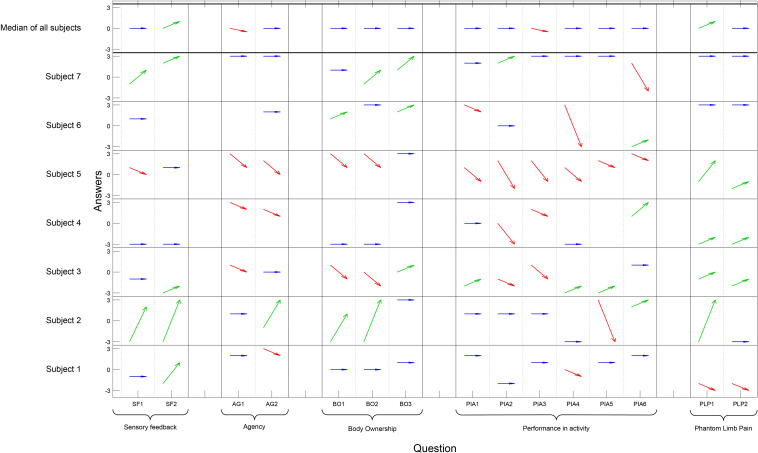
On the *x*-axis each question has a column and the questions are grouped according to concept. The *y*-axis presents each participant and the Likert scale. The answers pre- and post- are illustrated with an arrow for each question and participant; an improvement is illustrated by a green arrow, an impairment by a red arrow, and if there was no change the arrow is blue (Question PLP1–PLP2 for homogeneity in the table, a high value [3] indicates less pain). The six control questions are not presented in the figure. Wilcoxon signed rank test showed a significant positive change regarding Sensory feedback (*p* = 0.031), a significant negative change regarding Agency (*p* = 0.023), and Performance in Activity (*p* = 0.007). No significant changes were seen in the other concept categories in the questionnaire.

The responses in the questionnaire varied a lot among the participants, and also among the questions (Figure 3). Four out of seven participants experienced less agency compared to when using their normal prosthesis. There was a significant negative change regarding Agency [pre/post median 2 and 0 (*p* = 0.023)], and Performance in activity [pre/post median 1 and 0 (*p* = 0.007)], but there was a significant improvement regarding Sensory feedback (pre/post median −1 and 0 [*p* = 0.031]). Four of the five individuals with PLP reported a decrease of pain when using the sensory feedback prosthesis (pre/post median −2 and 0.5) but the change was not significant (Figure 3). One reported an increase of pain and associated it with the tight socket fit of the prosthesis. The six control questions were visually analyzed and all answers were on the far disagree-side of the scale, diverged from the rest of the answers, and filled their purpose and was removed prior analysis.

#### Directed Content Analysis

##### Sensory feedback from the prosthesis

The air-mediated system gave a sensory feedback when the fingertips of the prosthesis were compressed and at the same time the corresponding area of the PHM was stimulated by pressure. Someone described it as a tingling feeling. However, the feedback was experienced as too weak and it was difficult to feel small objects with the fingertips. A distinct change in the pressure, as when grasping or releasing, was needed for the user to notice the feedback. When using the prosthesis in heavy manual work, the experience from the feedback vanished, but when releasing the grip the change in pressure was noticed. The feedback seemed to be dependent on which activity the prosthesis was used in, and the feedback could disappear due to other disturbing impressions that come with the use of a myoelectric prosthesis, and was easily disturbed by muscle activity. One of the participants found it hard to feel the feedback at all, possibly due to scarring on the stump. It was expressed that the more the prosthesis was used, the better the feedback. A nice feeling as “scratching” the fingers could be experienced when pressing the bubbles of the prosthesis fingers. The feedback was perceived as the pressure really stimulated the corresponding finger. The feedback was distinct when touching the prosthetic fingertips with the other fingers on their own hand, in contrast to when using the prosthesis actively. A kind of feedback was experienced also in the regular prosthesis, e.g., vibrations in the socket when using the prosthesis. When comparing this to the sensory feedback in the test prosthesis it was described as a completely different feeling. The feedback from the test prosthesis was more like a real sensational experience which could be surprising. Even if the sensation was weak and not beneficial for practical use the experience of sensory feedback from the prosthesis was strongly expressed as feeling the touch in the prosthetic finger. They very much appreciated feeling the feedback, and it was also expressed that the experience of the sensory feedback was so good that it was desired in the regular prosthesis.

*“When touching the bubbles (fingertips) I got full feedback from all of them, when I don’t have any other load on the arm. When I sit in a relaxed position, then it is very distinct.”* (Id 7)

*“It is this*…*feeling, when I grasp something it really feels like I grasp it!”* (Id 2)

*“I have really bad sensibility on the stump and a lot of scars, so it was difficult to feel the feedback.”* (Id 4)

##### Agency

The feeling of agency was expressed in different terms. The participants expressed that even though they liked the sensory feedback, the feeling of agency did not change much; they controlled the prosthesis as they normally did. For better grip control a stronger feedback was desired. The experience of agency with the regular prosthesis was compared with wearing a thick oven glove, to be able to control the movement but lack the normal sensibility.

*“I like the feeling. When there is a sensory response the movement is more natural.”* (Id 3)

*“If the feedback would have been stronger I may have had more utility regarding controlling the grip.”* (Id 6)

##### Body ownership

It was expressed that the sensory feedback contributed to a strong feeling of completeness. The prosthesis felt like a part of them and this was a really pleasant feeling. It was described in quite an abstract way as if they could feel something that did not exist but which was still strongly linked to them. The prosthesis with feedback was used in situations when participants would not normally wear a prosthesis and when it was not used actively. To enjoy the feeling of touch from the prosthetic hand the participants touched the prosthesis with the other hand when relaxing, e.g., when watching TV. It was expressed that the connection to the test prosthesis became stronger because of the feeling of touch and that was a reason for wearing the prosthesis for a longer time than required.

*“It is a big feeling. I feel complete!”* (Id 2)

*“*…*it feels more like a part of me. I don’t know how to express it, but it feels good!”* (Id 7)

##### Performance in activity

The participants reported that they used the prosthesis when performing normal tasks, e.g., at work, the gym, and at home. They did everything they usually did with their regular prosthesis, such as working at the computer, cooking, cleaning, shopping, gardening, driving, biking, etc. Someone mentioned that the prosthesis was removed in heavy activities due to the risk of damaging and puncturing the silicon bubbles, e.g., when carrying heavy things, or at the gym. In this prototype prosthesis, the design was a hinder in some situations, e.g., it was hard to handle small nuts and to hold cutlery because of the soft bubbles/sensors on the fingertips of the prosthesis.

*“The sensory feedback has not helped me so much in the practical performance, so to speak, but I have a feeling*…*”* (Id 6)

*“I cannot feel small objects.”* (Id 1)

##### Suggestions for improvements

The participants felt that the sensory feedback needs to be improved. The feedback was not strong enough to be detected during muscle contractions when controlling the electrodes of the myoelectric function, and the feedback was too weak in active grips but sufficient when touching the fingers passively. The prototype prosthesis was experienced as clumsier than their normal prosthesis. Smaller bubbles on the fingertips were suggested, to give a more precise feedback when handling small objects, but also to enable a more distinct grip. The bubbles were considered too soft and it was hard to hold small objects and use force simultaneously. It was also noted that for the feeling of pleasantness from the touch of all fingers, the fingers should be represented separately. For grasping control the feedback areas might be larger. The appearance of the prototype prosthesis was not satisfactory. The bubbles/sensors on the fingertips looked oversized and the air-tubes were visual on the dorsal side of the prototype prosthetic hand. Some of the participants were not confident being around people with the prosthesis on and wore it only at home because of its appearance. An improvement of the aesthetics was therefore requested.

*“I want a more defined pressure, not so fuzzy. It is a soft feeling and it is too slow. I want more of an impact, something more distinct.”* (Id 5)

*“The air bulbs might be developed a bit, in terms of getting stronger feedback in the grip.* (Id 6)

### Objective Results

**FIGURE 4:**
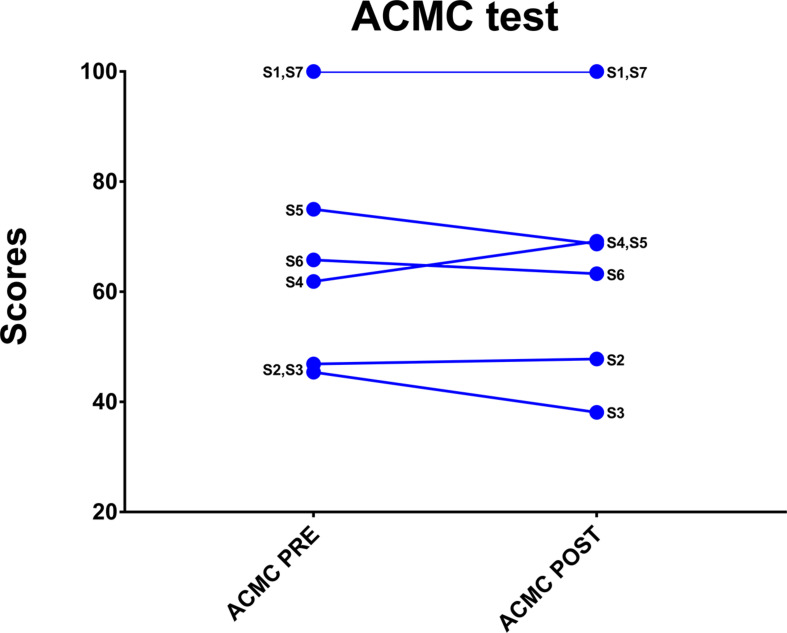
The ACMC-scores for each participant at pre-tests and follow-up.

The objective outcomes were analyzed descriptively. The ACMC showed a median score of 65.8 (range 45.4 – 100) in pre-tests and 68.5 (range 38.1 – 100) at follow-up (Figure 4.). Three individuals showed no change (1, 2, 7); three individuals had a worsened performance (3, 5, 6); and only one individual showed an improved performance. The Proprioceptive pointing task (Figure 2) when the participants were asked to mark on a ruler where they estimate the prosthetic index finger was at median deviation of 1.5 cm (range −10 – 4) in pre-test and −2 (range −9.5 – 1) at follow-up. When marking where they experienced their phantom index finger the distance relative to the prosthetic index finger was −14 cm (range −20 – −5) proximal to the prosthetic index finger in pre-tests and −13.5 (range −17.5 – 3) proximal to the prosthetic index finger at follow-up. The Wilcoxon signed rank test showed no differences between pretests and follow-up (not significant) regarding ACMC and Proprioceptive pointing task.

## Discussion

The use of a non-invasive sensory feedback system in daily activities over 4 weeks resulted in positive subjective experiences linked to body ownership and experiences of sensory feedback from the prosthesis, but did not improve the performance with the prosthesis as rated by the ACMC and self-rated performance on the questionnaire. Three participants showed unchanged performance, three showed deteriorated performance, and one showed improved performance.

To achieve a broad understanding of the evaluation of the prosthesis with sensory feedback, multiple methods were used. Qualitative analyses of interviews were performed as well as quantitative measures by use of a questionnaire and objective measurements, so-called method triangulation. In the analysis and report of results, as in the conclusions drawn, equal focus was on both the quantitative and the qualitative results. In the interviews, it was expressed that the sensory feedback was experienced as real touch which contributed to a feeling of completeness, linked to body ownership of the prosthesis. However, the quantitative results did not verify the qualitative results regarding body ownership. Either there were no changes or the quantitative measurements were not responsive enough to measure change over time. Another explanation may be that when people express their thoughts more freely as in the interview situation, a subtler in-depth answer is achieved which can be difficult to capture in a questionnaire. We cannot say which of the quantitative or the qualitative results have the most impact (Murray, 2009). According to Patton (1999), it should be expected to come to assorted results when using method triangulation. Instead, when qualitative and quantitative measures are used together, it can emphasize the result from different perspectives.

The experience that our limbs actually are a part of our own body is a feeling that is generated by sensory and visual feedback (Tsakiris et al., 2007). The non-invasive sensory feedback could promote the integration of the hand prosthesis in the bodily self and enhance the acceptance of the prosthesis. When acceptance is high, this may also affect the performance in a positive way (D’Alonzo et al., 2015; Imaizumi et al., 2016; Beckerle et al., 2018). Markovic et al. (2018a) show that regardless of improvement in performance the participants reported a positive experience of the feedback and rated an increased embodiment of the prosthesis (Markovic et al., 2018a). In our study, it was expressed in the interviews that the prosthesis was worn also in situations when not using it in practice, as when watching a movie, just because of the pleasantness of experiencing the touch through the prosthesis. If the prosthesis was used and experienced only as a tool one would assume that the prosthesis would be taken off in such situations. The qualitative positive experience of body ownership of the prosthesis due to the sensory feedback was the most important result. In previous studies using the rubber hand illusion experimental setup (Botvinick and Cohen, 1998), amputees experienced a hand prosthesis with a robotic appearance like a part of their own body (Ehrsson et al., 2008).

Phantom limb pain was alleviated in four of the five participants with PLP in our study, and reported that the sensory feedback affected PLP in a positive way in the questionnaire. Similar results have earlier been reported with significantly decreased PLP when sensory feedback is added to a prosthetic hand (Dietrich et al., 2012; Page et al., 2018; Petrini et al., 2018). Restored balance between afferent and efferent signaling, which is the case when sensory feedback is present in a hand prosthesis, can be one explanation (Flor et al., 2006; Vaso et al., 2014). However, the effect on PLP should be further investigated.

It has been suggested that sensory feedback could improve the functioning and performance with prostheses. This hypothesis is based on the knowledge of the importance of sensibility for motor function and the ability to use one’s hands (Gardner and Johnson, 2013). However, the importance of sensory feedback in hand prostheses is debated. Some studies support the hypothesis and have shown improved control of grasping force when sensory feedback is added (Witteveen et al., 2015; Clemente et al., 2016). On the other hand, Markovic et al. (2018a) showed, in a recent study, that sensory feedback was beneficial only when it came to complex tasks such as relocating clothespins and turning blocks. In simpler tasks, such as the “Box and Block Task,” the sensory feedback was not that important for performance. When naive prosthesis users (able-bodied) learned to control a myoelectric prosthesis, they showed relative good skills also without added feedback, just learning the muscle control needed for controlling the EMG-electrodes. However, when adding feedback sources, such as sound and vision, there was an improvement of the control (Markovic et al., 2018b). Similar results were shown in amputees, where task performance improved only by learning motor control. However, when vibrotactile feedback was added, task performance was further improved especially in complex tasks (Markovic et al., 2018a). Our results, however, suggest that sensory feedback does not improve the performance in the chosen activities. The worse performance that was experienced in some cases was probably due to changed socket fitting, the adjustment of the EMG electrodes, or bulky bulbs in the prosthetic fingertips. These changes may have altered the reliability of the prosthesis. The participants in our study were experienced prosthesis users, and they had over several years learned how to control their own prosthetic hand, probably relying on several sources of feedback such as vision, hearing, and proprioception (Wijk and Carlsson, 2015). Another possibility could be too weak feedback that was “masked” in muscle contractions.

The participants expressed that the best quality of the sensory feedback was achieved when they touched the prosthesis’ fingers themselves. They thought it was pleasant, comfortable, or even fantastic to touch the prosthesis. They felt the touch as if it was their own fingers being touched, even if it was a single modality feedback. It was during these moments the feeling of ownership could occur or the attachment to the prosthesis became stronger. Beckerle et al. (2018) discusses three aspects of touch: social, affective, and self-touch. They highlight the concept of self-touch because of its importance for both establishing representation of our bodies as infants and for maintaining this representation throughout life (Bremner and Spence, 2017). Self-touch is also significant for the experience of body ownership (Hara et al., 2015; Beckerle et al., 2018), and in our study the participants’ expressions confirms that.

The sensory feedback system used in this study is a simple and low-tech concept, but still both modality and somatotopically matched. However, the prototype system has potential for improvement. The feedback should be stronger or adjustable for each individual, which could be possible if the system used electric pressure sensors and, for example, electric motors as feedback actuators, which means that the feedback can be adjusted individually. The benefit of our system is that it is integrated in the prosthesis glove and the socket, and that it is not dependent on the type of prosthesis. The location of sensors in the glove was individualized in our study based on which fingers were represented in the PHM. In future versions, the prosthesis glove can be further developed for each individual and with desired location of both sensors and actuators, by making the system flexible. It can be applied to different types of prostheses and since the system is air-mediated and not dependent on the prosthesis design it is also suitable for, for example, body-powered or cosmetic prostheses. In a myoelectric prosthesis, there may be advances regarding practical use and the feeling of grip control, but as our result shows the sensory feedback has importance in emotional dimensions, thus regardless of the type of prosthesis. Another point that needs to be taken into account in the development of hand prostheses is the robustness and reliability to the prosthesis. If a prosthetic hand often breaks, or needs frequent and extensive service, it may be considered too much bother and end in rejection.

### Methodological Considerations

The system was integrated in a glove and a socket, not bigger than their respective size in a regular myoelectric hand prosthesis. Evaluation was made in everyday life with focus on activity in an environment that was relevant and meaningful for every participant. It includes complex tasks in an uncontrolled environment where the participant may not have complete focus on the prosthesis and the feedback. Most research in the area of sensory feedback is made in a laboratory environment, where tests and most evaluations are standardized into a few simple grip tests (Clemente et al., 2016; Schiefer et al., 2018; Mastinu et al., 2019). The prosthesis used in this study is still at a prototype stage, with cables on the outside, and because of that some participants experienced the prosthesis as less cosmetically appealing and chose to use it only at home. On the other hand, some of the seven participants used it full days, including at work, in sports activities, and domestics. We did not have control over the time when the prosthesis was used, or what the participants did when wearing the prosthesis; instead we had to rely on the information given. Maybe wearing the prosthesis 2 h/day, which was suggested as minimum, in the 4-week test period is not enough to change behavior and capacity in experienced users.

Minor discrepancies in the adjustment of the sensitivity of the EMG-electrodes in the new socket that was provided for the study could also affect the skillfulness. If the new socket of the test-prosthesis did not feel exactly like the regular one, this could be a disturbance in the use and performance with the prosthesis or even a pain trigger.

The studied group was small and heterogeneous. Almost all the participants were skilled prosthesis users, some of them for decades. If one has used a myoelectric prosthesis for several hours a day for many years, the capacity of controlling the grip and grasping of the prosthesis is very good. Markovic et al. (2018a) also claim that the feedback is not as important for experienced prosthesis users as for novel users. Regarding the qualitative analyses the low number of participants may also limit the possibility to achieve saturation of data. However, a low number of participants is a frequent problem in this research area, due to a low number of cases with transradial amputation and meeting the specific inclusion criteria.

All the interviews were carried out by the first author. Some of the participants had met the interviewer also at the clinic as a patient, which may have influenced the interview situation and affected the dependability of the results. It could be inhibitory for the respondents, but it may even deepen the interview and the narratives being shared.

To check for trustworthiness, specifically dependability, the first author and one co-author read and coded the interviews independently and by in-depth analysis and discussion interpreted the text together (investigator triangulation) (Patton, 1999; Carter et al., 2014). The trustworthiness was also achieved by including representative quotations from the participants, making the interpretation transparent for the reader. Constant confirming and clarifying information during the interviews assured confirmability. The focus was consistently on the text to reduce the risk of over-interpretation. Transferability is limited but a thorough description of the participants and the study context is presented (Graneheim and Lundman, 2004).

## Conclusion

The participants expressed that this non-invasive, somatotopically and modality matched sensory feedback system has positive qualities regarding feeling of body ownership and experienced sensory feedback from the prosthesis. In addition, it may alleviate phantom pain. However, performance with the prosthesis was not improved. The positive experience of the sensory feedback was highly expressed, and the users felt complete while wearing the prosthesis. The technical solution presented here has to be seen as a prototype with potential for improvements. The aesthetics need to be better so it can be used without shyness in social contexts. The silicon bulbs (sensors) on the fingertips were quite large which made them bulky in fine manipulation. Participants wished for a stronger or more distinct pressure, which should be taken into account in further development of the actuators. The silicon bulbs were also in some situations experienced as too soft, making it hard to hold small objects, or to get a distinct grip. It is of outmost importance to have close communication with the users in future developmental work.

## Implications for Rehabilitation

1. The positive (qualitative) experience of body ownership of the prosthesis due to the sensory feedback was the most important result.
2. The participants expressed that the best quality of the sensory feedback was achieved when they touched the prosthesis’ fingers themselves.
3. There is a need to evaluate different features of hand prosthesis, and it is of importance to do it close to the users in a meaningful environment.
4. The phantom hand map (PHM) offers a possibility to transfer sensory, non-invasively, information from the prosthesis to the user.

## Data Availability Statement

The datasets generated for this study are available on request to the corresponding author.

## Ethics Statement

The studies involving human participants were reviewed and approved by Regional Ethics Review Board in Lund, Sweden. The patients/participants provided their written informed consent to participate in this study.

## Author Contributions

BR, UW, AB, and CA participated in the design of the study. UW conducted the interviews and the tests. UW and IC read and analyzed the data from the interviews. The content in the predefined categories were then confirmed by all co-authors. UW, CA, and BR analyzed the data from the self-report questionnaire and objective data. UW wrote the manuscript. IC, CA, AB, and BR revised and approved the manuscript. All authors contributed to the article and approved the submitted version.

## Conflict of Interest

The authors declare that the research was conducted in the absence of any commercial or financial relationships that could be construed as a potential conflict of interest.

## Footnotes

1 http://acmc.se/

## Abbreviations

ACMC: The Assessment of Capacity for Myoelectric Control
EMG: electromyographic
PHM: phantom hand map
PLP: phantom limb pain.
